# Applicability of the ICD-11 proposal for PTSD: a comparison of prevalence and comorbidity rates with the DSM-IV PTSD classification in two post-conflict samples

**DOI:** 10.3402/ejpt.v6.27070

**Published:** 2015-05-18

**Authors:** Nadine Stammel, Eva M. Abbing, Carina Heeke, Christine Knaevelsrud

**Affiliations:** 1 Department of Clinical Psychology and Psychotherapy, Freie Universität Berlin, Berlin, Germany; 2 Center for Torture Victims, Berlin, Germany

**Keywords:** PTSD, trauma, psychiatric diagnosis, DSM, ICD, prevalence, comorbidity

## Abstract

**Background:**

The World Health Organization recently proposed significant changes to the posttraumatic stress disorder (PTSD) diagnostic criteria in the 11th edition of the International Classification of Diseases (ICD-11).

**Objective:**

The present study investigated the impact of these changes in two different post-conflict samples.

**Method:**

Prevalence and rates of concurrent depression and anxiety, socio-demographic characteristics, and indicators of clinical severity according to ICD-11 in 1,075 Cambodian and 453 Colombian civilians exposed to civil war and genocide were compared to those according to the Diagnostic and Statistical Manual of Mental Disorders, Fourth Edition (DSM-IV).

**Results:**

Results indicated significantly lower prevalence rates under the ICD-11 proposal (8.1% Cambodian sample and 44.4% Colombian sample) compared to the DSM-IV (11.2% Cambodian sample and 55.0% Colombian sample). Participants meeting a PTSD diagnosis only under the ICD-11 proposal had significantly lower rates of concurrent depression and a lower concurrent total score (depression and anxiety) compared to participants meeting only DSM-IV diagnostic criteria. There were no significant differences in socio-demographic characteristics and indicators of clinical severity between these two groups.

**Conclusions:**

The lower prevalence of PTSD according to the ICD-11 proposal in our samples of persons exposed to a high number of traumatic events may counter criticism of previous PTSD classifications to overuse the PTSD diagnosis in populations exposed to extreme stressors. Also another goal, to better distinguish PTSD from comorbid disorders could be supported with our data.

Three decades have now passed since the first definition of posttraumatic stress disorder (PTSD) in the third edition of the Diagnostic and Statistical Manual of Mental Disorders (DSM; American Psychiatric Association [APA], 1980). Yet, the diagnosis is still the subject of considerable debate (Brewin, Lanius, Novac, Schnyder, & Galea, 2009). Recently, a proposal for the 11th edition of the International Classification of Diseases (ICD-11) was published with significant changes in the PTSD classification (Maercker et al., 2013). The ICD-11 will be relevant especially for low- to middle-income countries (Maercker et al., 2013). The Mental Health Gap Action Programme (mhGAP), which is aimed at improving mental health care in low- to middle-income countries, as well as World Health Organization projects dealing with mental health care in the context of humanitarian crises, will use the ICD-11 classification. For this reason, the developers of the ICD-11 placed great emphasis on its applicability for health professionals of different disciplines and across different clinical settings and regions of the world.

The ICD-11 reformulation was intended to respond to criticism of previous PTSD classifications based on the ICD-10 and DSM-IV, which have been criticized on three main grounds (Maercker et al., 2013). First, there has been concern about the overlap of PTSD symptoms with symptoms of depression and other anxiety disorders, such as loss of interest or irritability. A second major criticism of previous PTSD classifications concerns their potential overuse in populations exposed to extreme stressors such as natural and man-made disasters (Maercker et al., 2013; Summerfield, 2001). Critics argue that PTSD symptoms cannot be distinguished from common stress reactions in these populations. Especially, the ICD-10 has been criticized for not including a requirement of functional impairment, making the distinction between normal and pathological reactions to traumatic events difficult. A third criticism focused on the trauma criterion, which defines the range of events that can be considered traumatic (Brewin et al., 2009). It has been debated to what extent this criterion is too broadly defined, so that it includes almost any human experience and risks being meaningless, or too narrowly defined.

The ICD-11 reformulation for PTSD is based on a proposal put forward by Brewin et al. (2009). A complete overview of the proposal is given in the Appendix. The classification is composed of three criteria (re-experiencing, avoidance, and perceived current threat), each of them containing two symptoms (Maercker et al., 2013). For the diagnosis of PTSD, at least one symptom of each criterion needs to be present for the period of several weeks after the exposure to an “extremely threatening or horrific event or series of events” (Maercker et al., 2013, p. 200). The most striking difference to previous classifications is the small number of qualifying symptoms in the ICD-11 proposal. In contrast to the DSM-IV, which comprises 17 symptoms, the ICD-11 proposal has only six qualifying symptoms. Symptoms that overlapped with other disorders or have been shown to be less important were removed (Brewin et al., 2009). The symptom reduction was also intended to simplify the diagnostic process especially in low-resourced, non-English-speaking settings, consistent with the objective of the ICD-11 to be globally applicable (Brewin, 2013). This approach is in great contrast to the newest version of the DSM, the DSM-5 (APA, 2013). As the ICD-11 proposal includes only a minimum number of core symptoms, the DSM-5 working group opted for the opposite strategy and gave a comprehensive description of 20 symptoms typically encountered. A second difference compared to previous classifications is the formulation of the trauma criterion. The statement from the ICD-10 that the traumatic event is “likely to cause pervasive distress in almost everyone” was removed in the ICD-11 proposal in order to refocus PTSD on the core symptoms (Brewin et al., 2009; Maercker et al., 2013). Furthermore, this change reflects previous studies such as the DSM-IV Field Trial, which suggested that changing the definition of trauma had no impact on PTSD prevalence rates (Kilpatrick et al., 1998). A third difference to the ICD-10 classification is the inclusion of a diagnostic requirement for functional impairment. In this manner, the ICD-11 working group increased the threshold for a PTSD diagnosis and responded to the criticism of its overuse in population exposed to extreme stressors.

Thus far, four studies have investigated the impact of the proposed ICD-11 criteria and yielded mixed results. A study by Van Emmerik and Kamphuis (2011) investigated the impact of the original proposal put forward by Brewin et al. (2009) on PTSD prevalence and comorbidity rates based on a sample of 170 treatment-seeking civilian trauma survivors. The classification proposed by Brewin et al. (2009) is similar to the current ICD-11 proposal, except that it did not include a trauma criterion. They found no change in PTSD prevalence rates compared to the DSM-IV, although 13% of participants gained a PTSD diagnosis under the Brewin criteria and 13% lost their diagnostic status. Rates of comorbidity with anxiety disorders and depression were consistently lower under the Brewin criteria in comparison to the DSM-IV, but these differences did not reach significance. Morina, Van Emmerik, Andrews and Brewin (2014) investigated the differences in the prevalence of PTSD as well as comorbid major depression and anxiety disorders in two samples of 560 Kosovar civilian war survivors and 142 British war veterans. They did not find differences in PTSD prevalence rates between DSM-IV and ICD-11, but less comorbidity with major depression when applying the ICD-11 criteria. Two recent studies investigated the effects of the ICD-11 proposal on prevalence and comorbidity rates and other indicators of clinical severity in comparison to the ICD-10, the DSM-IV, and the DSM-5 (O'Donnell et al., 2014; Stein et al., 2014). The study of Stein et al. (2014) was based on the World Mental Health Surveys that assessed 23,936 participants with reported lifetime traumatic events. Results indicated similar prevalence rates under the proposed ICD-11 classification compared to the DSM-IV. Comorbidity rates with fear and distress disorders were marginally lower under the proposed ICD-11 criteria compared to the DSM-IV. Similarly, PTSD severity, as measured by severe distress or impairment and high suicidality, was lower under the proposed ICD-11 compared to the DSM-IV. The study by O'Donnell et al. (2014) was based on 510 injury patients. Results showed lower prevalence rates under the ICD-11 compared to the DSM-IV. Comorbidity with depression was significantly lower under the ICD-11 compared to DSM-IV. Indicators of clinical severity, such as the proportion of participants with high disability, were lower under the ICD-11 compared to DSM-IV.

## Objective

The purpose of the present study was to replicate and extend previous studies by comparing the proposed ICD-11 classification of PTSD with the DSM-IV classification based on two different post-conflict samples of persons exposed to genocide and civil war. Our samples consisted of participants from two different cultural backgrounds exposed to a very high number of war-related traumatic events. The two samples can be regarded as representative for populations exposed to collective violence. Based on the results of previous studies (Morina et al., 2014; O'Donnell et al., 2014; Stein et al., 2014), we did not expect that the proposed changes in the PTSD classification lead to different results in the PTSD prevalence rates in both samples. Similar to previous studies, the main focus of our study was to investigate the changes in PTSD prevalence, PTSD caseness, and concurrent depression and anxiety relative to the DSM-IV classification. We were also interested in whether both classification systems have similar risk factors in terms of socio-demographic and trauma-related characteristics. Furthermore, we analyzed whether participants who either lost or gained a PTSD diagnosis under the ICD-11 proposal have a similar clinical severity in terms of symptom severity and suicidality.

## Methods

### Participants and procedure

The data for the study were obtained from two different surveys conducted in Cambodia and Colombia. The survey in Cambodia included victims of the Khmer Rouge regime and was conducted in 19 different provinces between October 2008 and May 2009 (Stammel, Burchert, Taing, Bockers, & Knaeveslrud, 2010). Following the suggestion by Wirtz (2004) to remove cases with more than 30% missing data, two participants were excluded from data analysis. The final sample consisted of 1,075 participants with an average age of 56.3 years (SD=10.3), ranging between 35 and 98 years. The majority of the participants (61.7%, *n*=663) were female, 66.2% (*n*=712) were married and 27.9% (*n*=300) were widowed. Most of the participants (89.2%, *n*=959) were ethnic Khmer and 92.2% (*n*=991) described themselves as Buddhists. The average time spent in school was 4.0 years (SD=3.6). All participants experienced traumatic events or witnessed others suffering from traumatic events such as lack of food or water (95.0%, *n*=1,021), forced labor (92.1%, *n=*990), and forced separation from family members (79.7%, *n=*857). On average, participants experienced 13.6 traumatic events (SD=3.9). A complete overview of the sampling procedure and other socio-demographic information can be found in Stammel et al. (2010).

The second survey was conducted in Colombia between September and December 2012 (Stammel, Heeke, Diaz Gomez, Ziegler, & Knaevelsrud, 2012). One participant was excluded from data analysis due to more than 30% missing data on the PTSD, depression, and anxiety symptom items. All 453 participants of the final sample were victims of internal displacement in the context of the Colombian armed conflict. The sample consisted of 264 female (58.3%) and 189 male participants with a mean age of 47.9 years (SD=13.1), ranging from 18 to 85 years. The majority of the participants was either married (26.9%, *n*=122) or in a relationship (34.2%, *n*=155), 9.9% (*n*=45) were widowed and 10.2% (*n*=42) were divorced. The mean time spent at school was 5.7 (SD=4.0) years. Most of the participants identified themselves as Mestizo (51.2%, *n*=232), 15.0% (*n*=68) as Afrocolombian, and 6.6% (*n*=30) belonged to the indigenous population. Concerning their religious faith, 59.2% (*n*=270) of the participants indicated that they were of Catholic faith and 21.0% (*n*=95) of Christian faith. All the participants had either experienced or witnessed traumatic events. The most frequent traumatic experiences were being threatened with violence and death (77.7%, *n*=352), war experiences (77.5%, *n*=351), and 64.5% (*n*=292) witnessed the murder of one or several strangers. On average, participants experienced 9.64 traumatic events (SD=3.92). For a complete overview of the sampling procedure and other socio-demographic information, see Stammel et al. (2012).

In both samples, data were collected prior to the proposal for ICD-11, thus with a different scope as of the current article. The aim of both studies was to learn about the effects of genocide and civil war on mental health as well as about attitudes toward the ongoing processes of reparations in both countries at the time of the studies. In both studies, participants were informed about the study's content and objectives, the duration of the interviews, the voluntary nature of their participation, the principles of confidentiality and anonymity, and their right to refuse to answer any question. The interviews were conducted by native-speaking interviewers. The interviewers followed a training of 2 weeks prior to the survey. They were supervised by experienced psychologists. The interviews were structured and face-to-face and conducted in Khmer or Spanish language. The studies were approved by the University Konstanz Review Board (Cambodia) and the Freie Universität Berlin Review Board (Colombia).

### Measures

In both samples, the presence of PTSD symptoms was determined using the Posttraumatic Stress Disorder Checklist-Civilian Version (PCL-C; Weathers, Litz, Huska, & Keane, 1994). The PCL-C is based on the DSM-IV diagnostic criteria for PTSD (APA, 2000). It assesses 17 symptoms of PTSD on a five-point Likert scale (1=“not at all” to 5=“extremely”). Participants were asked to rate the symptoms experienced in the past month. In the Colombian study, items assessing the functional impairment criterion of the DSM-IV (criterion F) were added from the Posttraumatic Diagnostic Scale (PDS; Foa, Cashman, Jaycox & Perry, 1997). The PCL-C has shown good performance in a variety of cultural settings (Miles, Marshall, & Schell, 2008; Tol et al., 2007; Vera-Villarroel, Zych, Celis-Atenas, Córdova-Rubio, & Buela-Casal, 2011). The alpha reliability of the 17-item scale was 0.90 in the Colombian sample and 0.88 in the Cambodian sample, indicating that the scale had very good internal consistency. A symptom rated with 3 (“moderately”) or higher on the PCL-C was classified as present (Andrykowski, Cordova, Studts, & Miller, 1998). For a diagnosis of PTSD based on the DSM-IV, a participant needed to experience at least one symptom of the re-experiencing criterion B, three or more symptoms of avoidance criterion C, and two or more symptoms of the hyperarousal criterion D for the period of at least 1 month. In addition, a PTSD diagnosis under DSM-IV required the experience of a traumatic event (criterion A1) and specific emotional reactions during the trauma (criterion A2), as well as significant functional impairment.

In order to assess the presence of PTSD under the proposed ICD-11 diagnostic system, only six symptoms of the PCL-C were included, closely approximating the six symptoms of the ICD-11 and grouped into three core features. Items 2 (“Repeated disturbing dreams of a stressful experience from the past”) and 3 (“Suddenly acting or feeling as if a stressful experience were happening again”) correspond to the first core feature “re-experiencing” of the ICD-11. Items 6 (“Avoid thinking about or talking about a stressful experience from the past or avoid having feelings related to it”) and 7 (“Avoid activities or situations because they remind you of a stressful experience from the past”) correspond to the second core feature “avoidance.” Whereas items 16 (“Being ‘super alert’ or watchful on guard”) and 17 (“Feeling jumpy or easily startled”) correspond to the third core feature “hypervigilance or enhanced startle reactions.” For PTSD to be present, one symptom within each core feature was required as well as functional impairment and a symptom duration of several weeks. In the Colombian sample, the stressor criteria A1 and A2 were not coded separately and, therefore, only information on whether or not the stressor criterion was met can be provided. In the Cambodian sample, the functional impairment criterion was not included in the data collection.

The Hopkins Symptom Checklist-25 (HSCL-25) was used to measure symptoms of depression and anxiety experienced in the past week (Derogatis, Lipman, Rickels, Uhlenhuth, & Covi, 1974). It examines 10 symptoms of anxiety and 15 symptoms related to depression on a scale ranging from 1 (“not at all”) to 4 (“extremely”). The scale has shown good performance in populations with different cultural backgrounds (Mollica, Wyshak, De Marneffe, Khuon, & Lavelle, 1987; Silove et al., 2007). The depression score was the average of the 15 depression items. We used a cut-off score of 1.75 or above on the depression subscale to indicate “caseness” (Nettelbladt, Hansson, Stefansson, Borgquist, & Nordström, 1993). The same cut-off score was applied for the anxiety subscale. The alpha reliability of the depression subscale was 0.89 in the Cambodian sample and 0.88 in the Colombian sample. The alpha reliability of the anxiety subscale was 0.88 in the Cambodian sample and 0.89 in the Colombian sample.

Traumatic events were assessed using an adjusted checklist based on the Harvard Trauma Questionnaire (Mollica et al., 1992) and the PDS (Foa et al., 1997). We included seven traumatic events specific to the Khmer rouge system (e.g., “forced marriage” and “forced labor”) and one specific to the Colombian conflict (“get disappeared”). In the Colombian sample, a total of 23 traumatic events were assessed and 29 in the Cambodian sample. Suicidality was assessed with the item “thoughts of ending your life” of the HSCL-25, ranging from 1 (“not at all”) to 4 (“extremely”).

### Analyses

The following analyses were performed separately for the Cambodian and Colombian samples. Analyses of prevalence, concurrent depression and anxiety, socio-demographic characteristics, and indicators of clinical severity were conducted using SPSS 21.0.

Prior to our analyses, we performed a missing value analysis using SPSS version 21.0. In both samples, the percentage of missing data was small (Cambodian sample: 0.854% and Colombian sample: 0.211%). Expectation–maximization algorithm was used to impute a single new data set with no missing values. This method has the disadvantage that standard errors tend to be smaller, which may lead to biased results in hypothesis testing (Graham, 2009; Von Hippel, 2004). However, due to the small percentage of missing data and based on the assumption that the data were missing completely at random, we imputed the data only once.

#### Analysis of PTSD prevalence

We first calculated the proportions of participants meeting the criteria for a PTSD diagnosis under either DSM-IV or ICD-11. The analyses were based on the outcomes of the PCL-C. We calculated the number and proportion of participants who (1) met the PTSD diagnoses under the DSM-IV (*total DSM-IV group*), (2) met the proposed ICD-11 criteria (*total ICD-11 group*), (3) met the diagnosis under the ICD-11, but not under the DSM-IV (*ICD-11-only group*), (4) met the diagnosis under the DSM-IV, but not under the proposed ICD-11 criteria (*DSM-IV-only group*), and (5) participants who did not meet the diagnostic criteria under either diagnostic system. To evaluate the differences in PTSD prevalence, two-tailed binomial approximation *z*-tests for proportions were calculated.

#### Analysis of concurrent depression and anxiety

We then calculated the rates of concurrent depression and anxiety. We calculated the total score (depression and anxiety) as well as the depression and anxiety subscales separately. We used Chi-square tests to test the differences in concurrent depression and anxiety rates between the ICD-11-only group and the DSM-IV-only group. In the Colombian sample, sample sizes were too small in the ICD-11-only group, so that descriptive statistics are reported for interest, but statistical tests were not performed.

#### Differences in socio-demographic and trauma-related characteristics

Socio-demographic and trauma-related characteristics were assessed using means and standard deviations or frequencies and proportions, as applicable. We tested differences in age, sex, and number of traumatic events between the ICD-11-only and the DSM-IV-only groups. We performed a Chi-square test for the difference in sex, and unrelated *t*-tests to test the differences in age and number of traumatic events.

#### Differences in PTSD clinical severity

We compared the differences in PTSD severity between the ICD-11-only and the DSM-IV-only groups. PTSD severity was based on the PCL-C items ranging from 1 “not at all” to 5 “extremely.” For the PTSD severity score based on the DSM-IV, all 17 items were summed up and standardized. For the severity score based on the ICD-11, the six items approximating the ICD-11 proposal were summed up and standardized. We performed unrelated *t*-tests to test differences in PTSD severity between the two groups. Suicidality was assessed with the item “thoughts of ending your life” of the HSCL-25. Differences in suicidality between the DSM-IV-only and the ICD-11-only groups were tested with unrelated *t*-tests.

## Results

### Prevalence

Overall, PTSD prevalence rates were higher when the DSM-IV diagnostic criteria were applied compared to the proposed ICD-11 criteria set. As shown in Table 1, in the Cambodian sample, significantly more participants met the DSM-IV PTSD criteria compared to the ICD-11 criteria set, *t=*3.477, *p=*0.001. In total, 8.5% of participants changed their diagnostic status when the ICD-11 criteria were used. The level of agreement between both diagnostic systems was high (91.5% agreement, *κ*=0.515, *p<*0.001). Also in the Colombian sample, relatively more participants received a diagnosis under the DSM-IV criteria compared to the ICD-11 criteria set, *z=*6.143, df=452, *p<*0.001 (Table 2). In total, 14.6% of participants lost or gained a PTSD diagnosis under ICD-11, and the agreement between both diagnostic systems was substantial (85.4% agreement, *κ*=0.712, *p<*0.001).

**Table 1:** Proportion of participants meeting DSM-IV and proposed ICD-11 criteria for PTSD in the Cambodian sample

					Changes in diagnosis under proposed ICD-11 criteria					
	
	Total DSM-IV		Total ICD-11		No longer present		Unchanged		Newly present	
					
	*n*	%	*n*	%	*n*	%	*n*	%	*n*	%
Stressor criterion A	1,047	97.4	1,075	100						
A1. Traumatic event	1,075	100	1,075	100						
A2. Emotional response	1,047	97.4								
Re-experiencing criterion B	566	52.7	237	22.0	329	30.6	746	69.4	0	0.0
B1. Distressing recollections	367	34.1								
B2. Distressing dreams	171	15.9	171	15.9						
B3. Flashbacks	126	11.7	126	11.7						
B4. Psychological reactivity	382	35.5								
B5. Physiological reactivity	380	35.3								
Avoidance criterion C	159	14.8	265	24.7	35	3.3	899	83.6	141	13.1
C1. Avoiding internal reminders	191	17.8	191	17.8						
C2. Avoiding external reminders	158	14.7	158	14.7						
C3. Specific amnesia	133	12.4								
C4. Diminished interest	211	19.6								
C5. Detachment	139	12.9								
C6. Restricted affect	90	8.4								
C7. Foreshortened future	203	18.9								
Hyperarousal criterion D	487	45.3	413	38.4	119	11.1	911	84.7	45	4.2
D1. Difficulty sleeping	506	47.1								
D2. Irritability	353	32.8								
D3. Difficulty concentrating	325	30.2								
D4. Hypervigilance	153	14.2	153	14.2						
D5. Exaggerated startle response	350	32.6	350	32.6						
Duration criterion	970	90.2	970	90.2						
Impairment criterion^a^	–	–	–	–						
PTSD diagnosis	120	11.2	87	8.1	62	5.8	984	91.5	29	2.7

PTSD=posttraumatic stress disorder; total DSM-IV=participants meeting diagnostic criteria for PTSD under DSM-IV; total ICD-11=participants meeting diagnostic criteria for PTSD under the proposed ICD-11 classification.

a The impairment variable was not included in the data.

**Table 2:** Proportion of participants meeting DSM-IV and proposed ICD-11 criteria for PTSD in the Colombian sample

					Changes in diagnosis under proposed ICD-11 criteria					
	
	Total DSM-IV		Total ICD-11		No longer present		Unchanged		Newly present	
					
	*n*	%	*n*	%	*n*	%	*n*	%	*n*	%
Stressor criterion A^a^	453	100	453	100						
A1. Traumatic event^a^										
A2. Emotional response^a^										
Re-experiencing criterion B	409	90.3	310	68.4	99	21.9	354	78.1	0	0.0
B1. Distressing recollections	292	64.5								
B2. Distressing dreams	247	54.5	247	54.5						
B3. Flashbacks	260	57.4	260	57.4						
B4. Psychological reactivity	328	72.4								
B5. Physiological reactivity	291	64.2								
Avoidance criterion C	330	72.8	355	78.4	26	5.7	376	83.0	51	11.3
C1. Avoiding internal reminders	302	66.7	302	66.7						
C2. Avoiding external reminders	272	60.0	272	60.0						
C3. Specific amnesia	181	40.0								
C4. Diminished interest	280	61.8								
C5. Detachment	274	60.5								
C6. Restricted affect	186	41.1								
C7. Foreshortened future	277	61.1								
Hyperarousal criterion D	343	75.7	333	73.5	31	6.8	401	88.5	21	4.6
D1. Difficulty sleeping	271	59.8								
D2. Irritability	237	52.3								
D3. Difficulty concentrating	263	58.1								
D4. Hypervigilance	262	57.8	262	57.8						
D5. Exaggerated startle response	278	61.4	278	61.4						
Duration criterion	432	95.4	432	95.4						
Impairment criterion	337	74.4	337	74.4						
PTSD diagnosis	249	55.0	201	44.4	57	12.6	387	85.4	9	2.0

PTSD=posttraumatic stress disorder; total DSM-IV=participants meeting diagnostic criteria for PTSD under DSM-IV; total ICD-11=participants meeting diagnostic criteria for PTSD under the proposed ICD-11 classification.

a The stressor criteria A1 and A2 were not inquired separately. There is only information on whether the criterion A was met or not.

Next, we compared the proportion of participants meeting the three PTSD criteria (re-experiencing, avoidance, and hyperarousal) between the total ICD-11 and the total DSM-IV group. In both samples, the ICD-11 re-experiencing criterion was more stringent than the DSM-IV criterion (Colombian sample: *z*=11.243, *p<*0.001; Cambodian sample: *z*=21.764, *p*<0.001). This was also true for the hyperarousal criterion, though the difference only reached significance in the Cambodian sample (*z=*5.868, *p<*0.001) and not in the Colombian sample (*z=*1.388, *p=*0.166). The opposite effect was found for the avoidance criterion, which was more stringent in the DSM-IV classification (Colombian sample: *z=*−2.872, *p=*0.004; Cambodian sample: *z=*−8.235, *p<*0.001).

### Concurrent depression and anxiety

In the Cambodian sample, 322 participants (30.0%) were above the threshold of depression (*M*=1.603, SD=0.563), 396 participants (36.8%) were above the threshold for anxiety (*M*=1.681, SD=0.646), and 268 participants (24.9%) were above the cut-offs of both anxiety and depression. In the Colombian sample, 307 participants (67.8%) were above the threshold of depression (*M*=2.103, SD=0.597), 268 (59.2%) above anxiety (*M*=2.050, SD=0.716), and 240 participants (53.0%) above the threshold for both anxiety and depression (Tables 3 and 4).

**Table 3:** Proportion of participants with concurrent depression or other anxiety disorders among Cambodian participants meeting DSM-IV or ICD-11 criteria for PTSD

	Total DSM-IV (*n=*120)		Total ICD-11 (*n=*87)		DSM-IV only (*n=*62)		ICD-11 only (*n=*29)		ICD-11 only vs. DSM-IV only		
					
	*n*	(%)	*n*	(%)	*n*	(%)	*n*	(%)	OR	95% CI	*p*
Depression	104	86.7	69	79.3	49	79.0	14	48.3	0.25	0.10, 0.64	<0.001
Anxiety	101	84.2	73	83.9	48	77.4	20	69.0	0.65	0.24, 1.74	0.39
Total score	93	77.5	63	72.4	42	67.7	12	41.4	0.34	0.14, 0.84	0.02

PTSD=posttraumatic stress disorder; total DSM-IV=participants meeting diagnostic criteria for PTSD under DSM-IV; total ICD-11=participants meeting diagnostic criteria for PTSD under the proposed ICD-11 classification; DSM-IV only=participants meeting PTSD criteria only under DSM-IV; ICD-11 only=participants meeting PTSD criteria only under ICD-11; OR=odds ratios; CI=confidence interval.

**Table 4:** Proportion of participants with concurrent depression or other anxiety disorders among Colombian participants meeting DSM-IV or ICD-11 criteria for PTSD

	Total DSM-IV (*n=*249)		Total ICD-11 (*n=*201)		DSM-IV only (*n=*57)		ICD-11 only (*n=*9)	
				
	*n*	(%)	*n*	(%)	*n*	(%)	*n*	(%)
Depression	221	88.8	179	89.1	48	84.2	6	55.6
Anxiety	190	76.3	158	78.6	37	64.9	5	66.7
Total score	180	72.3	151	75.1	34	59.6	5	55.6

PTSD=posttraumatic stress disorder; total DSM-IV=participants meeting diagnostic criteria for PTSD under DSM-IV; total ICD-11=participants meeting diagnostic criteria for PTSD under the proposed ICD-11 classification; DSM-IV only=participants meeting PTSD criteria only under DSM-IV; ICD-11 only=participants meeting PTSD criteria only under ICD-11.

We compared the rates of concurrent depression and anxiety symptoms between participants who gained or lost their diagnostic status under the ICD-11 (ICD-11-only group and DSM-IV-only group, respectively). In the Cambodian sample, the rates of concurrent depression (*χ* ^2^=8.774, df=1, *p<*0.01) and of the total score (*χ* ^2^=5.691, df=1, *p=*0.017) were significantly higher in the DSM-IV-only group compared to the ICD-11-only group. Rates of concurrent anxiety were also higher in the DSM-IV-only group, though the difference was not significant (*χ* ^2^=0.748, df=1, *p*=0.387).

In the Colombian sample, the rates of concurrent anxiety were similar between the ICD-11-only and the DSM-IV-only group, whereas rates of concurrent depression and the total score were higher among participants meeting the DSM-IV criteria only. Statistical tests were not performed due to small sample sizes.

To sum up, in both samples,the rates of concurrent depression and the total score of the HSCL-25 were higher among participants meeting the DSM-IV only, compared to those meeting only the ICD-11 criteria set. These differences reached significance in the Cambodian sample, but could not be tested in the Colombian sample. Rates of concurrent anxiety did not show significant differences between the two groups.

### Socio-demographic and trauma-related characteristics

In the following, we examined whether participants in the ICD-11-only and the DSM-IV-only group differed with regard to socio-demographic and trauma-related characteristics. Overall, there were no significant differences with respect to sex, age, and number of traumatic events between the two groups. In the Colombian sample (Table 5), the sample size of the ICD-11-only group (*n=*9) was very small and an examination of the descriptive statistics suggested no significant differences between the two groups. The following analyses were, therefore, only performed for the Cambodian sample (Table 6). No significant differences were found with regard to sex (*χ* ^2^=0.748, df=1, *p*=0.387), age ( *t=*−0.481, df=89, *p=*0.632), and number of traumatic events (*t*=−1.414, df=89, *p*=0.161).

**Table 5:** Socio-demographic and trauma-related characteristics and indicators of clinical severity of participants meeting DSM-IV and ICD-11 criteria for PTSD in the Colombian sample

	Total DSM-IV (*n=*249)		Total ICD-11 (*n=*201)		DSM-IV only (*n=*57)		ICD-11 only (*n=*9)	
				
Categorical variables	*N*	%	*n*	%	*n*	%	*n*	%
Gender (Female)	157	63.1	125	62.2	37	64.9	5	62.8
Continuous variables	*M*	SD	*M*	SD	*M*	SD	*M*	SD

Age	48.24	12.63	47.29	12.54	51.18	11.84	45.44	6.50
Number of traumatic experiences	10.60	3.57	10.62	3.72	10.47	3.19	10.22	4.76
Symptom severity	0.64	0.65	0.76	0.64	0.18	0.41	0.18	0.41
Suicidality	1.49	0.88	1.57	0.93	1.19	0.58	1.44	0.88

PTSD=posttraumatic stress disorder; total DSM-IV=participants meeting diagnostic criteria for PTSD under DSM-IV; total ICD-11=participants meeting diagnostic criteria for PTSD under the proposed ICD-11 classification; DSM-IV only=participants meeting PTSD criteria only under DSM-IV; ICD-11 only=participants meeting PTSD criteria only under ICD-11. The symptom severity score were standardized. The suicidality scores were based on a scale from 1=not at all to 4=extremely. The symptom severity scores were standardized based on the 17 symptoms of the DSM-IV for the DSM-IV total and the DSM-IV-only groups. For the ICD-11 total and ICD-only group, they were standardized based on the six symptoms of the ICD-11 proposal.

**Table 6:** Socio-demographic and trauma-related characteristics and indicators of clinical severity of participants meeting DSM-IV and ICD-11 criteria for PTSD in the Cambodian sample

	Total DSM-IV (*n=*120)		Total ICD-11 (*n=*87)		DSM-IV only (*n=*62)		ICD-11 only (*n=*29)	
				
Categorical variables	*n*	%	*n*	%	*n*	%	*n*	%
Gender (Female)	97	80.8	69	79.3	48	77.4	20	69.0
Continuous variables	*M*	SD	*M*	SD	*M*	SD	*M*	SD

Age	55.22	9.68	55.89	9.16	54.32	10.75	55.48	10.69
Number of traumatic experiences	12.64	3.94	13.38	4.20	11.83	3.73	13.10	4.62
Symptom severity	1.89	0.81	2.08	0.81	1.53	0.59	1.67	0.59
Suicidality	1.48	0.94	1.39	0.87	1.48	0.94	1.24	0.69

PTSD=posttraumatic stress disorder; total DSM-IV=participants meeting diagnostic criteria for PTSD under DSM-IV; total ICD-11=participants meeting diagnostic criteria for PTSD under the proposed ICD-11 classification; DSM-IV only=participants meeting PTSD criteria only under DSM-IV; ICD-11 only=participants meeting PTSD criteria only under ICD-11. The suicidality scores were based on a scale from 1=not at all to 4=extremely. The symptom severity scores were standardized based on the 17 symptoms of the DSM-IV for the DSM-IV total and the DSM-IV-only groups. For the ICD-11 total and ICD-only group they were standardized based on the six symptoms of the ICD-11 proposal.

### Indicators of clinical severity

Next, we compared PTSD severity and suicidality between the DSM-IV-only group and the ICD-11-only group. In the Cambodian sample, symptom severity did not differ significantly between the two groups (*t*=−1.094, df=89, *p*=0.277). Similarly, we found no significant differences with regard to suicidality. Because the variances for the two groups were significantly unequal (*F*=6.239, *p=*0.014), a *t*-test for unequal variances was used and found to be non-significant (*t=*1.388, df=72,413, *p*=0.169). Overall, participants meeting only ICD-11 criteria did not differ significantly from participants meeting only DSM-IV criteria with regard to socio-demographic characteristics and indicators of clinical severity.

## Discussion

The main purpose of this study was to explore how the proposed ICD-11 criteria for PTSD affect prevalence and concurrent rates of depression and anxiety relative to DSM-IV diagnostic criteria in two different non-western post-conflict samples exposed to a high number of traumatic events. We also investigated differences in socio-demographic and trauma-related characteristics and indicators of clinical severity.

### PTSD prevalence

Overall, a substantial proportion (Cambodian sample: 14.6%; Colombian sample: 8.5%) of participants lost or gained a PTSD diagnosis under ICD-11 criteria, indicating that each classification system identifies individuals, which the other fails to identify. In the current study, the DSM-IV classification showed significantly higher prevalence rates of PTSD compared to the ICD-11 proposal in both samples. This finding is consistent with the results of O'Donnell et al. (2014), but inconsistent with the studies by Van Emmerik and Kamphuis (2011), Morina et al. (2014) as well as Stein et al. (2014), which showed no differences in PTSD prevalence rates. Differences among the samples might explain the inconsistent results: In contrast to the studies by Van Emmerik and Kamphuis (2011) and Stein et al. (2014), the present study was based on participants exposed to a very high number of mostly war-related traumatic experiences. Nearly all of our participants met criterion A2 (emotional response) of the DSM-IV compared to only 61% in the study by Van Emmerik and Kamphuis (2011). This criterion was not included in the diagnostic criteria of the ICD-11 proposal. Whereas it had no impact on prevalence rates in our study, it is likely to have lowered PTSD prevalence rates under the DSM-IV in the studies by Van Emmerik and Kamphuis (2011) and Stein et al. (2014), thereby resulting in more similar prevalence rates under both classification systems. In contrast, relatively more participants (80%) met the stressor criterion A2 in the study by O'Donnell et al. (2014), so that its impact on DSM-IV prevalence rates was reduced. The study by Morina et al. (2014) did not provide information on the percentage of participants meeting criterion A2.

Our study suggests that the removal of qualifying symptoms alone reduces PTSD prevalence rates under the ICD-11 proposal compared to the DSM-IV. The A2 criterion in the DSM-IV might have countered this effect in previous studies. Thus, the overall lower prevalence rate under the ICD-11 compared to the DSM-IV in our study suggests that the ICD-11 proposal might cause lower prevalence rates especially in populations exposed to extreme stressors (where nearly everybody would meet criterion A2). The results, therefore, support the aim of the ICD-11 proposal to reduce the overuse of the PTSD diagnosis in populations exposed to man-made or natural disaster. It should be investigated, however, to what extent individuals who would lose a PTSD diagnosis under the ICD-11 criteria would stop qualifying for psychological treatment despite high levels of suffering and functional impairment related to traumatic experiences. Furthermore, it should be considered that someone who loses a PTSD diagnosis might still meet criteria for another disorder, such as depression.

The effect of the symptom reduction on the number of participants meeting each of the three PTSD symptom criteria was similar to previous studies (O'Donnell et al., 2014; Van Emmerik & Kamphuis, 2011). The re-experiencing and hyperarousal criteria were less stringent under DSM-IV compared to the ICD-11 proposal, whereas the avoidance criterion was more stringent under DSM-IV. This is not surprising, as the probability of meeting at least one out of five symptoms in the DSM-IV re-experiencing criterion is higher than the probability to meet one out of two symptoms in the ICD-11 re-experiencing criterion. Furthermore, one of the symptoms excluded in the ICD-11 proposal was intrusive memories, which is a common symptom of many disorders (Brewin et al., 2009). The less stringent avoidance criterion under the ICD-11 proposal is possibly due to the reduced threshold in the ICD-11 proposal requiring one out of two symptoms compared to three out of seven symptoms in the DSM-IV. The two avoidance symptoms included in the ICD-11 proposal were also among those most commonly endorsed by our participants.

### Concurrent depression and anxiety

One main goal of the ICD-11 proposal for PTSD was to better distinguish PTSD from common comorbid disorders such as depression and anxiety disorders. When comparing participants who only met ICD-11 criteria with those only meeting the DSM-IV PTSD diagnosis in the Cambodian sample (ICD-11-only group and DSM-IV-only group, respectively), the rates of concurrent depression and the total score (depression and anxiety) were significantly lower under the ICD-11 proposal, whereas the rates of concurrent anxiety did not differ significantly. The descriptive statistics in the Colombian sample were similar, but no significance testing could be performed due to small sample size. The results are in line with the study by Morina et al. (2014), who also found significantly lower comorbidity rates with depression. In contrast, the study by Van Emmerik and Kamphuis (2011) showed no differences in comorbidity rates. Differences in the statistical analyses might account for the inconsistent results: The study by Van Emmerik and Kamphuis (2011) compared all participants meeting DSM-IV criteria with all participants meeting ICD-11 criteria (total DSM-IV group and total ICD-11 group, respectively) using binomial approximation *z*-tests. The overlap between these two groups is likely to have lowered the power of these tests. Taken together, our study suggests that the proposed ICD-11 PTSD diagnosis is better able to distinguish PTSD from depression.

### Socio-demographic and trauma-related characteristics and indicators of clinical severity

Overall, there were no significant differences in socio-demographic and trauma-related characteristics between participants meeting only DSM-IV or the proposed ICD-11 diagnostic criteria. This is in line with the study by Stein et al. (2014) and indicates that both classification systems share similar underlying risk factors. In line with this, participants meeting only DSM-IV or ICD-11 diagnostic criteria were also similar in symptom severity and suicidality, suggesting that each classification system misses individuals with different symptom patterns, despite a similar level of clinical severity. Our results regarding suicidality are in contrast to the study by Stein et al. (2014), which showed substantially lower suicidal behavior in patients meeting only ICD-11 diagnostic criteria compared to patients meeting only DSM-IV criteria. One reason for this might be that our study measured suicidal ideation instead of suicidal behavior and used one item only, instead of a questionnaire as was done in the study by Stein et al. (2014).

### Limitations of the study

There were several limitations to this study that should be addressed in future research. First, we relied on data that were collected previous to the publication of the ICD-11 proposal, so that the proposed ICD-11 classification could only be approximated. The results might, therefore, be imprecise. However, the reported previous studies faced the same problems. Furthermore, our study did not use thresholds for the levels of suicidality and symptom severity, which would have given an indication to what extent each system omits individuals with significant clinical severity. Due to the reliance on previously collected data, we could not make the more relevant comparison between the ICD-11 proposal and the recently published DSM-5 PTSD diagnostic criteria. However, the studies by Stein et al. (2014) and O'Donnell et al. (2014) suggest that PTSD prevalence and comorbidity rates do not differ significantly between the DSM-5 and DSM-IV classifications. Our study might, therefore, still give an indication on the effects of the proposed ICD-11 PTSD criteria relative to the DSM-5 in populations exposed to a high number of war experiences. Future research should, nevertheless, investigate to what extent these two classification systems lead to differences in prevalence, comorbidity, and disability, and whether they might favor different PTSD phenotypes, which may become an obstacle to the study and diagnosis of PTSD.

A major goal of the ICD-11 proposal was to improve clinical utility especially in resource-poor, non-English speaking settings. Therefore, it is important to study potential changes in PTSD diagnosis in such settings, as was done in the current study. More specific PTSD diagnosis in such settings might help to better distribute scarce resources by getting a more realistic image of their psychological strain and by improving the allocation of persons to specific treatments.

## Appendix

Proposed ICD-11 Classification PTSD is a disorder that develops following exposure to an extremely threatening or horrific event or series of events characterized by:

1. Re-experiencing the traumatic event(s) in the present in the form of vivid intrusive memories, flashbacks, or nightmares, with each episode of re-experiencing accompanied by fear or horror.
2. Avoidance of thoughts and memories of the event(s), or avoidance of activities or situations reminiscent of the event(s).
3. A state of perceived current threat in the form of excessive hypervigilance or enhanced startle reactions.

The symptoms must last for at least several weeks and cause significant impairment in personal, family, social, educational, occupational, or other important areas of functioning.
